# Early versus late initiation of renal replacement therapy for acute kidney injury in critically ill patients: A systematic review and meta-analysis

**DOI:** 10.1371/journal.pone.0223493

**Published:** 2019-10-24

**Authors:** Li Xiao, Lu Jia, Rongshan Li, Yu Zhang, Hongming Ji, Andrew Faramand

**Affiliations:** 1 Chengdu Women's and Children's Central Hospital, School of Medicine, University of Electronic Science and Technology of China,Chengdu, Sichuan, China; 2 Shanxi Provincial People’s Hospital, Taiyuan, China; 3 Affiliated Hospital of Chengdu University, Chengdu, Sichuan, China; 4 University of Pittsburgh Medical Center, Pittsburgh, Pennsylvania, United States of America; Azienda Ospedaliero Universitaria Careggi, ITALY

## Abstract

**Background:**

Acute kidney injury is associated with high mortality, and is the most frequent complication encountered in patients residing in the intensive care unit. Although renal replacement therapy (RRT) is the standard of care for acute kidney injury, the optimal timing for initiation is still unknown.

**Methods:**

We conducted a systemic review and meta-analysis of randomized controlled trials evaluating early versus late initiation of RRT in critically ill patients with acute kidney injury. We searched MEDLINE, Embase, and CENTRAL databases from inception to October 15, 2018. We screened studies and extracted data from published reported independently. The primary outcome was short-term mortality.

**Results:**

A total of 2242 patients were included from 11 trials. No statistically significant effect was found for early versus late initiation of RRT on short-term mortality (risk ratio [RR] 0.99, 95% CI 0.84–1.17, p = 0.93) or long-term mortality (RR 0.98, 95% CI 0.85–1.13, p = 0.76). There were also no statistically significant effects on ICU length of stay, hospital length of stay, recovery of renal function, and renal replacement therapy dependence. Early initiation of RRT decreased the risk of metabolic acidosis (RR 0.65, 95% CI 0.43–0.99, p = 0.04), but increased the risk of hypotension (RR 1.24, 95% CI 1.08–1.43, p = 0.003).

**Conclusions:**

In critically ill patients with acute kidney injury, early compared with late initiation of RRT is not associated with favorable mortality outcomes, although it appears to reduce the risk of metabolic acidosis.

## Introduction

Acute kidney injury (AKI) is a frequent complication in patients hospitalized in the intensive care unit (ICU) and is associated with high mortality.[1, 2] Real replacement therapy (RRT) is the cornerstone of the management of AKI, although it could also be associated with complications and adverse events.[3, 4] There is, however, an ongoing debate concerning when to initiate it. Earlier initiation of RRT may help with fluid and electrolyte balance, removal of uremic toxins, and in the prevention of complications (e.g., metabolic encephalopathy and gastric hemorrhage). However, early initiation of RRT may unnecessarily expose a subset of patients who spontaneously recover renal function to potential harm.[5]

Despite the physiologic rationale, randomized controlled trials (RCTs) examining the ideal time for initiation of renal replacement therapy for AKI remains controversial.[6–10] Recent meta-analyses[11–15] suggested that early initiation of RRT is not associated with lower mortality rates; however, those studies have been limited by inconsistency, imprecision, and the risk of publication bias. Since these reviews, several large RCTs [16, 17] on the topic have been published. Given the conflicting evidence of RCTs and the limitations of the previous meta-analyses, we conducted an updated systematic review and meta-analysis to investigate the effect on mortality of the timing of the initiation of RRT in patients with AKI.

## Methods

In this systematic review and meta-analysis, we followed the Preferred Reporting Items for Systematic Reviews and Meta-analyses (PRISMA) guidelines for the development of protocols and reporting of the study. **[18]** PRISMA checklist is reported in S2 File. Our protocol was registered at the international prospective register of systematic reviews (PROSPERO), number CRD42018115030.

### Information sources and search strategy

A medical librarian searched Medline, Embase, Cochrane Database of Systematic Reviews from inception to October 15, 2018. ClinicalTrials.gov and the World Health Organization International Clinical Trials Registry Platform were searched to identify ongoing trials. The reference lists of included studies were searched for additional studies. There were no restrictions on language. Details of the search strategy are presented in” material A in S1 File’”.

### Eligibility criteria

Studies were considered eligible if they met the following PICOS(Patients, Intervention, Comparison Outcomes, and Study design) criteria: (1) population: critically ill patients with AKI; (2) intervention: earlier initiation of RRT; (3) comparison intervention: late initiation of RRT; (4) outcome: at least one outcome of interest had to be reported; (5) study design: RCT. The definitions of the timing of initiation of RRT were at the discretion of the trials’ authors.

### Outcomes

The primary outcome was short-term mortality (within 31 days). The secondary outcomes were long-term mortality (60–180 days), ICU length of stay, hospital length of stay, renal replacement therapy dependence, renal function recover, adverse events (metabolic acidosis, hyperkalemia, bleeding event, infection, and hypotension).

### Study selection

After removal of duplicates, the title and abstracts of studies were independently screened in duplicate by two researchers (LX and YZ). The full texts of the remaining results were independently assessed in duplicate by the two researchers (LX and YZ) for inclusion based on predetermined criteria. Discrepancies were resolved by consensus among the study team.

### Data collection process

One research (LX) abstracted data into standardized collection forms and created tables for the evidence and outcomes. Another research (YZ) double checked the extracted data. Discrepancies were resolved by consensus among the study team. We contacted corresponding authors for unpublished information.

### Quality assessment

Two researchers (LX and YZ) independently assessed the risk of bias using the Cochrane Collaboration risk of bias tool. They evaluated the following domains: sequence generation, allocation concealment, blinding of patients and personnel, blinding of outcome assessors, incomplete outcome data, selective reporting, and other bias. Each domain was classified as either low, unclear, or high risk of bias. Discrepancies were resolved by consensus among the study team.

The grading of recommendations assessment, development, and evaluation (GRADE) approach was used to rate the quality of evidence and generate absolute estimates of effect for the outcomes to rate the quality of evidence for each outcome as high, moderate, low, or very low.[19]

### Data synthesis

Analyses were conducted using Review Manager version 5.3.3 (Cochrane Collaboration). Summary measures were pooled using random-effects models. For continuous outcomes, the mean difference (MD) with 95% CI was calculated. For dichotomous outcomes, we calculated the relative risk (RR) with 95% CI. Statistical significance testing was 2-sided and a P < .05 was considered statistically significant. Heterogeneity was assessed by I^2^ tests, with substantial heterogeneity defined as I^2^ greater than 50%.[20] We planned to assess publication bias using funnel plot inspection, Egger’s test, Begg’s test, and Harbord’test when 10 or more trials were pooled.

We performed multiple sensitivity analyses to investigate potential sources of inconsistency, including removal of individual trials at each time, removal of trials conducted before 2005, removal of trials including fewer than 100 patients, and removal of with non-low risk of bias of each domain.

We conducted prespecified subgroup analyses based on ICU type (surgical or mixed ICU), sepsis (yes or mixed), and type of RRT (continuous renal replacement therapy [CRRT], IHD [intermittent hemodialysis], or mixed). We also conducted meta-regression of control groups mortality to explore the effects of potential sources of heterogeneity.

A trial sequential analysis (TSA) using TSA software (version 0.9.5.9) was conducted to explore the minimum information size for the primary outcome. An optimal information size set to a two-sided 5% risk of a type I error, 20% risk of a type II error (power of 80%), and relative risk reduction of 20%.

## Results

### Study selection and study characteristics

**Fig 1:**
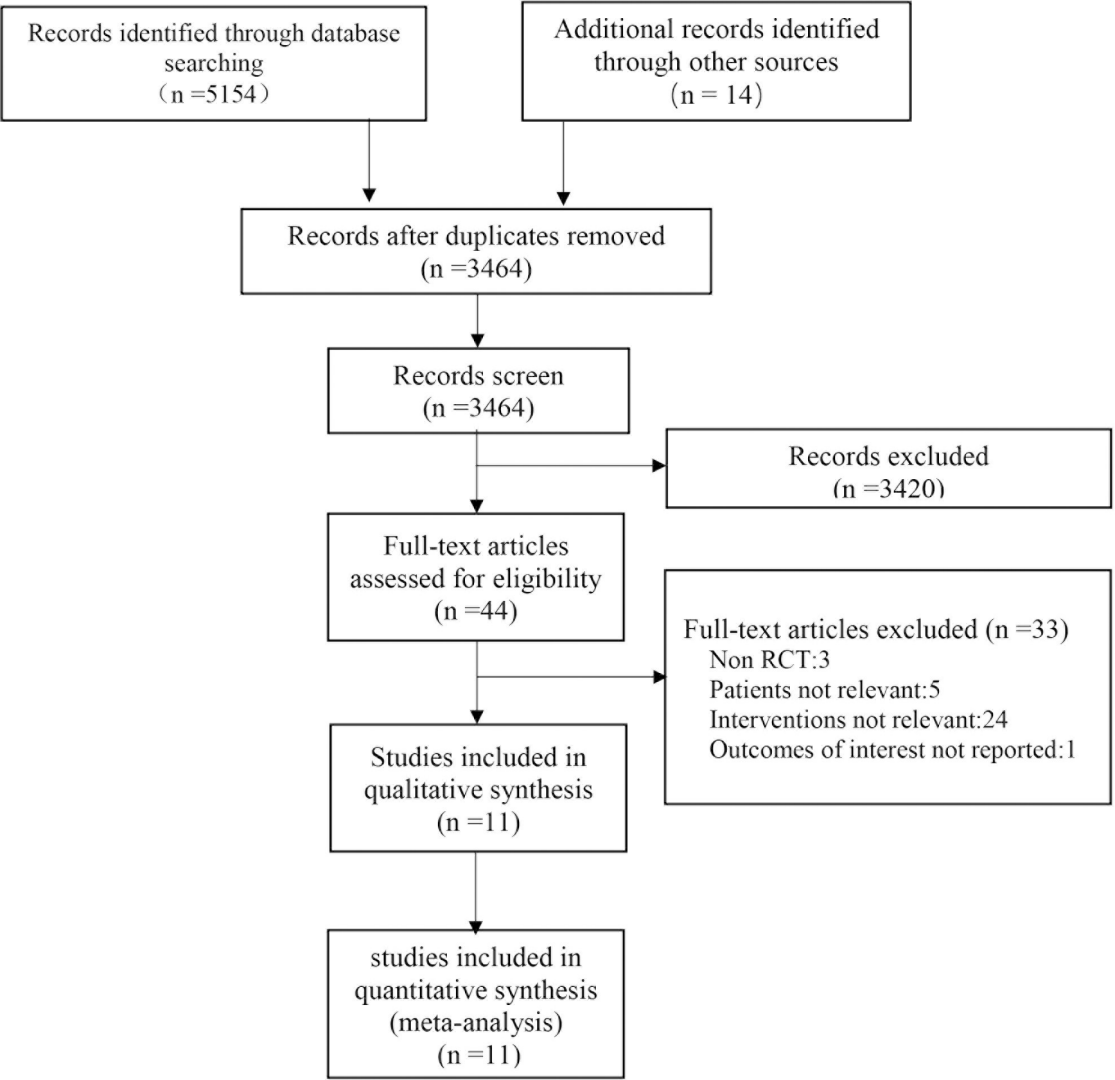
Study selection for inclusion in meta-analysis of the timing of initiation of renal replacement therapy.

The PRISMA flow diagram of the meta-analysis is shown in Fig 1. A total of 5,168 records were retrieved with 11 trials, including a total of 2242 participants, included in the study. [6–10, 16, 17, 21–24]

Table 1 and “Table A-B in S1 File” summarize the characteristics of the included trials. Population sizes ranged from 28 to 619, and five trials included more than 200 patients. Follow-up varied between 14 and 180 days.

**Table 1:** Characteristics of included studies.

Author	Year	Country	Population	Patients (No)	Mean Age (year)	Male (%)	Follow-up (days)
Bouman	2002	Netherlands	Surgery / medical	106	68	60	28
Durmaz	2003	Turkey	Cardiac Surgery	44	56	79	30
Sugahara	2004	Japan	Cardiac Surgery	28	65	64	14
Payen	2009	France	Medical/ Surgery	76	58	71	28
Jamale	2013	India	Medical	208	43	68	90
Combes	2015	France	Cardiac Surgery	224	59	79	90
Wald	2015	Canada	Medical/ Surgery	100	63	72	90
Gaudry	2016	France	Medical/ Surgery	619	66	65	60
Zarbock	2016	Germany	Surgery	231	67	63	90
Barbar	2018	France.	Medical	477	69	61	180
Lumlertgul	2018	Thailand	Medical	118	67	49	28

Risk of bias of included trials is shown in “Figure A-B in S1 File”. The Cochrane Collaboration’s tool indicated that all trials were adjudicated as high risk of bias because treating clinicians were not blinded to treatment allocation and outcome assessment. Table 2 summarizes the findings of all outcomes.

**Table 2:** Summary of findings and strength of evidence in trials comparing early vs late initiation of RRT.

Outcome	No. of patients (Studies)	Relative effect (95% CI)	I^2^	Absolute effect estimates (per 1000)			Quality
				Late	Early	Difference	
Mortality short term (≤31 days)	2207 (11)	RR 0.99 (0.84 to 1.17)	44%	392	392	0 (−59 to 67)	High
Mortality long term (60–180 days)	1662 (5)	**RR 0.98** (0.85 to 1.13)	42%	497	487	−10 (−75 to 65)	High
Length of stay in ICU	1658 (6)	MD 0.06 (-1.11 to 1.22)	0%	0.06 (-1.11 to 1.22)			High
Length of stay in hospital	1602 (6)	MD -1.09 (-3.53 to 1.36)	25%	-1.09 (-3.53 to 1.36)			High
Renal function recovery	1579 (9)	**RR 1.02** (0.97 to 1.07)	32%	549	560	11(−16 to 38)	High
Renal replacement therapy dependence	1036 (8)	**RR 0.77** (0.49 to 1.21)	0%	71	55	-16 (−36 to 15)	Moderate^1^
Metabolic acidosis	964 (4)	**RR 0.65** (0.43 to 0.99)	10%	126	82	-44 (−72 to -1)	Moderate^1^
Hyperkalemia	1583 (5)	**RR 0.53** (0.26 to 1.09)	42%	63	33	-30 (−47 to 6)	Moderate^1^
Hypotension	1020 (4)	**RR 1.24** (1.08 to 1.43)	0%	275	341	66 (22 to 118)	High
Bleeding event	1872 (8)	**RR 0.95** (0.76 to 1.2)	10%	167	159	-8 (40 to 33)	Moderate^1^
Infection	1877 (8)	**RR 1.12** (0.83 to 1.53)	32%	152	170	18 (−26 to 80)	Moderate^1^

CI: Confidence interval; RR: Risk ratio; MD: Mean difference

^1^ imprecisions

### Primary outcome

**Fig 2:**
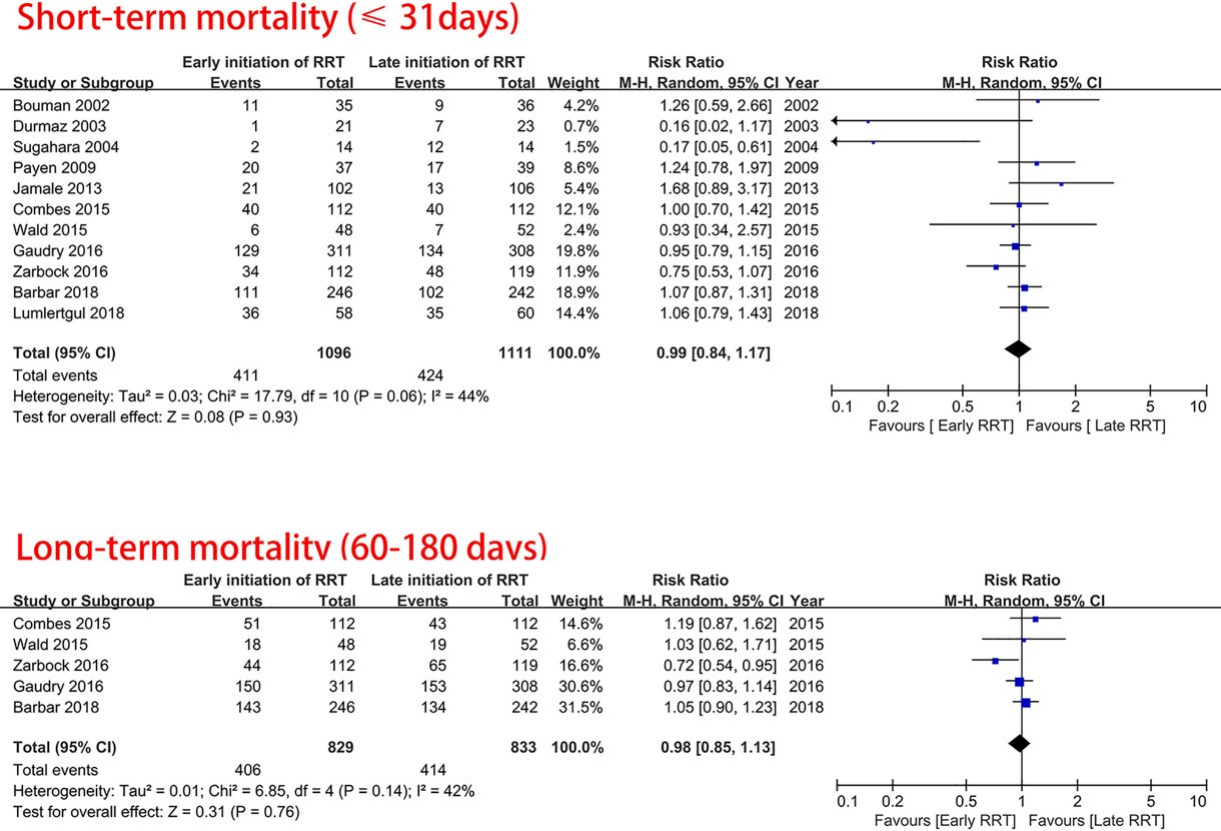
Association of early vs. delay of initiation of renal replacement therapy with short-term and long-term mortality.

The associations between the timing of initiation of RRT and mortality are shown in Fig 2. There were 11 trials including a total of 2207 participants with data available regarding short-term (≤31 days) mortality. The pooled RR for short-term mortality for early versus late initiation of RRT was 0.99 (95% CI 0.84–1.17, p = 0.93, I^2^ = 44%). The TSA for the primary outcome showed a required information size was not met. All sensitivity analyses were consistent with the main analysis (Table C in S1 File).

**Fig 3:**
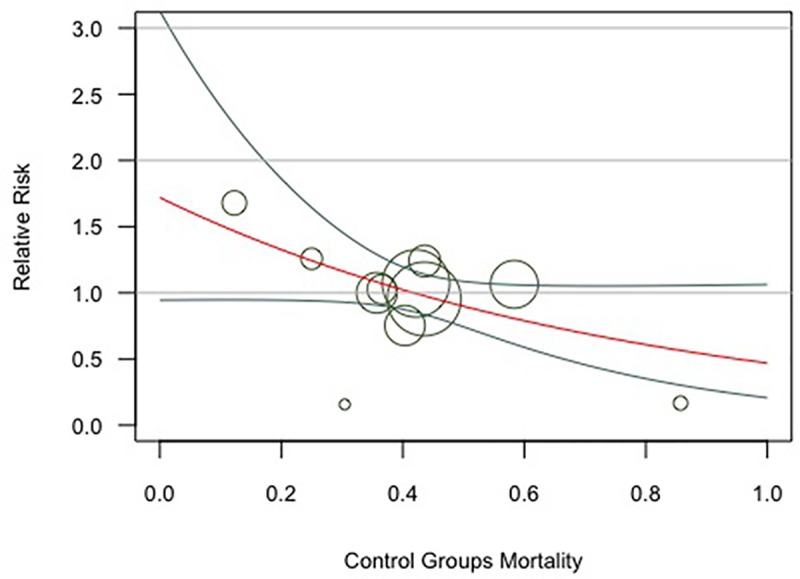
Meta-regression for short-term mortality outcome by control groups mortality (P = 0.06).

Multiple subgroup analyses did not demonstrate any credible subgroup effect (p > 0.05 for all outcomes, Table 3). Meta-regression examining the effect of control group mortality on short-term mortality did not demonstrate the possible sources of heterogeneity. (P = 0.06, Fig 3).

**Table 3:** Subgroup analysis of the effect of early initiation of RRT on short-term mortality.

Subgroup title	No. of studies	No. of patients	Risk Ratio, 95%CI	P
ICU type				
Surgical	4	899	0.66 [0.30, 1.48]	0.29
Mixed	11	1308	1.03 [0.90, 1.17]	
Sepsis				
Yes	2	564	1.10 [0.91, 1.32]	0.31
Mixed	9	1643	0.94 [0.75, 1.18]	
RRT type				
IHD	5	1018	0.97 [0.72, 1.31]	0.91
CRRT	2	252	0.62 [0.06, 6.53]	
Mixed	4	937	1.00 [0.86, 1.17]	
Overall	11	2207	0.99 [0.84, 1.17]	NA

CRRT, continuous renal replacement therapy; ICU, intensive care unit; IHD, intermittent hemodialysis; NA, not available

Funnel plot analysis suggested some asymmetry (Figure C in S1 File), and Egger test effects (p = 0.648), Begg’s test (p = 0.100), and Harbord’test (p = 0.302), detected no significant publication bias.

### Secondary outcomes

For long-term mortality, there were a total of 1622 participants from 1 trial[9] that reported mortality at 60 days, three trials[8, 10, 24] that reported mortality at 90 days, and one trial[16] that reported mortality at 180 days. The pooled RR for long-term mortality for early initiation of RRT compared to late initiation of RRT was 0.98 (95% CI 0.85–1.13, p = 0.76, I^2^ = 42%, Fig 2).

Pooled estimates suggest that different initiation of RRT result in similar ICU length of stay (MD 0.06 d, 95% CI -1.11 to 1.22, “Figure F in S1 File”) and hospital length of stay (MD -1.09 d, 95% CI -3.53 to 1.36, “Figure F in S1 File”). There was no difference in the incidence of renal function recovery (RR 1.02, 95% CI 0.97–1.07, “Figure G in S1 File) and renal replacement therapy dependence (RR 0.77, 95% CI 0.49–1.21, “Figure H in S1 File) between early versus late of initiation of RRT.

Early initiation of RRT decreased the risk of metabolic acidosis (RR 0.65, 95% CI 0.43–0.99, p = 0.04, “Figure I in S1 File”) but increased the risk of hypotension (RR 1.24, 95% CI 1.08–1.43, p = 0.003, “Figure J in S1 File”).

## Discussion

In this meta-analysis of 11 RCT involving 2242 patients with AKI, we found that early initiation of RRT did not affect either short-term or long-term mortality. Subgroup analyses did not identify credible effect modification in ICU type (surgical or mixed ICU), sepsis (yes or mixed), and RRT type. The early strategy for the initiation of RRT was not associated with hospital length of stay, ICU length of stay, the recovery of renal function, and renal replacement therapy dependence. Furthermore, early initiation of RRT decreased the risk of metabolic acidosis but increased the risk of hypotension.

### Principal findings and comparison with other studies

The most recent meta-analyses of RCTs have stated that early initiation of RRT is not associated with lower mortality rates. [11, 12, 14, 15, 25] It is consistent with our findings, but our study has narrower CIs and lower heterogeneity compared with the previous meta-analyses, which upgrades the quality of evidence from low to high. Furthermore, our study differs from previous studies in several ways. First, we separately reported mortality on the short-term and long-term basis. Second, this study included two recent trials[16, 17]. These data reinforced our findings, improved precision concerning the treatment effects of early initiation of RRT and decreased the heterogeneity of included trials. Third, we quantified two new findings, a reduced risk of metabolic acidosis and an increased risk of hypotension.

After this meta-analysis was submitted for initial review by PLOS ONE, Pasin et al. published an additional meta-analysis[26]. Their main conclusion was that early initiation of RRT in critically ill patients with AKI does not provide a clinically relevant advantage when compared with late initiation is like this study. However, this study found that early initiation of RRT decreased the risk of metabolic acidosis but increased the risk of hypotension. Moreover, the methodology of this study differs from that of Pasin et al.’s study. This study provided absolute as well as relative risks, explored the optimum sample size using TSA, and evaluated the quality of the evidence using GRADE approach.

Clinicians believe that earlier initiation of RRT confers a more prompt control of electrolyte and acid-base status, however, exposure to complications associated with RRT (eg, intradialytic hypotension, dysrhythmias, clearance of antibiotics) remains a concern.[5] This meta-analysis confirmed that metabolic acidosis is more frequent with late strategy whereas hemodynamic instability is more frequent with early strategy. We should weigh pros and cons more carefully to consider the clinical consequences of metabolic acidosis and hemodynamic instability. Though metabolic acidosis is more common than hypotension, hypotension has much more severe consequences than metabolic acidosis. Metabolic acidosis is very well tolerated and easy to correct with sodium bicarbonate administration[27], but hypotension is associated with increased mortality and a lower likelihood of renal recovery after AKI.[28]

### Strengths and limitations

Methodological strengths of this study include a comprehensive search for evidence, rigorous assessment of the quality of evidence, specification of both relative and absolute effects, application of the TSA to explore the minimum information size and use of meta-regression to explore the effect.

Our study also has limitations. First, this study is limited by clinical heterogeneities among the included trials. Those variabilities included eligibility criteria, RRT modalities, ICU setting, length of follow-up, definition of AKI, and definition of “earlier” RRT. For example, inclusion criteria across trials is mostly based on functional changes in serum creatinine and/or urine output, whereas Zarbock et al.’s trial used neutrophil gelatinase–associated lipocalin. These heterogeneities may have influenced the effect of early initiation of RRT. Second, all trials have a high risk of bias because of unblinded design. Hhowever, it was difficult to perform a double-blind method study. Third, this study separately assessed both short-term (within 31 days), and long-term (60–90 days) mortality. However, the effect of early initiation of RRT on morality longer than 180 days was unclear, because no trial reported mortality longer than 180 days.

### Applicability

Based on the findings of our study, high certainty evidence suggests no reduction in mortality with early initiation of RRT in AKI, which appear consistent across all studied subgroups. Thus, the adoption of early initiation of RRT in critically ill patients with AKI cannot be recommended for routine use unless further high quality and well-powered evidence shows the benefit of it. Additionally, an individual participant data meta-analysis would allow a more accurate assessment of the effect of earlier initiation of RRT on time-to-event and subgroup analysis.

### Conclusions

High-quality evidence suggests that early versus later initiation of RRT in critically ill patients with AKI had no significant effect on mortality, although it reduces the risk of metabolic acidosis. Based on the limitations of this study (e.g., the heterogeneity of entry criteria), ongoing trials should clarify (1) the special population to target; (2) the optimal defemination of early versus later initiation of RRT; (3) the heterogeneity of RRT deliverables. Further large RCTs are required to confirm the result of this meta-analysis.

## Supporting information

S1 File Material A: Search strategy for Medline; Table A: Definition of timing of RRT; Table B: Sensitivity analyses; Table: Sensitivity analyses; Figure A: Risk of bias summary; Figure B: Risk of bias graph; Figure C: Trial sequential analysis for short-term mortality; Figure D: Funnel plot for short-term mortality; Figure E: Forest plot for length of stay in hospital; Figure F: Forest plot for length of stay in ICU; Figure G: Forest plot for renal function recovery; Figure H: Forest plot for renal replacement therapy dependence; Figure I: Forest plot for metabolic acidosis; Figure J: Forest plot for hypotension.
(DOCX)

S2 File PRISMA 2009 checklist. (DOC)

## Abbreviations

AKI: Acute kidney injury
ICU: intensive care unit
RRT: renal replacement therapy
PRISMA: Preferred Reporting Items for Systematic Reviews and Meta-analyses
RCT: randomized controlled trial
PICOS: Patients, Intervention, Comparison Outcomes, and Study design
GRADE: the grading of recommendations assessment, development, and evaluation
RR: relative risk
TSA: trial sequential analysis
