# Supplementary material for: Early versus late initiation of renal replacement therapy for acute kidney injury in critically ill patients: A systematic review and meta-analysis
Source: PLoS One. 2019 Oct 24;14(10):e0223493. doi: 10.1371/journal.pone.0223493 (PMC6812871; doi:10.1371/journal.pone.0223493)

**Supporting information**

|  |  |
| --- | --- |
| material A: Search strategy for Medline |  |
| Table A: Definition of timing of RRT |  |
| Table B: Sensitivity analyses  Table C: Sensitivity analyses |  |
| Figure A: Risk of bias summary |  |
| Figure B: Risk of bias graph |  |
| Figure C: Trial sequential analysis for short-term mortality |  |
| Figure D: Funnel plot for short-term mortality |  |
| Figure E: Forest plot for length of stay in hospital |  |
| Figure F: Forest plot for length of stay in ICU |  |
| Figure G: Forest plot for renal function recovery |  |
| Figure H: Forest plot for renal replacement therapy dependence |  |
| Figure I: Forest plot for metabolic acidosis |  |
| Figure J: Forest plot for hypertension |  |

### **material A**: Search strategy for Medline

((((((((("Renal Dialysis"[Mesh]) OR "Dialysis"[Mesh]) OR "Peritoneal Dialysis"[Mesh]) OR "Kidneys, Artificial"[Mesh]) OR "Acute Kidney Injury/therapy"[Mesh])) AND "Acute Kidney Injury"[Mesh]))) OR (((((((((("Renal Dialysis"[Tiab]) OR "Dialysis"[Tiab]) OR "Peritoneal Dialysis"[Tiab]) OR "Kidneys, Artificial"[Tiab]) OR "Acute Kidney Injury/therapy"[Tiab])) AND "Acute Kidney Injury"[Tiab]))))) AND ((((randomized controlled trial[pt] OR clinical trial, phase iii[pt] OR clinical trial, phase iv[pt] OR clinicaltrials.gov[si] OR isrctn[si] OR randomized controlled trials as topic[mh]) OR (clinical trial[pt] AND (((single[tw] OR double[tw] OR doubleblind[tw] OR doubleblinded[tw] OR treble[tw] OR triple[tw]) AND (blind[tw] OR blinded[tw] OR mask[tw] OR masked[tw] OR masks[tw] OR sham[tw] OR shams[tw] OR dummy[tw])) OR (random[tw] OR randomise[tw] OR randomize[tw] OR randomised[tw] OR randomized[tw] OR rct[tw] OR rcts[tw] OR single-blind method[mh] OR double-blind method[mh] OR random allocation[mh]))) AND ((comparative study[pt] OR compare[tw] OR compares[tw] OR compared[tw] OR comparing[tw] OR comparison[tw] OR comparative[tw] OR effective[tw] OR effectiveness[tw] OR versus[ti] OR vs[ti]) OR (activities of daily living[mh] OR benefit[tw] OR benefits[tw] OR budgets[mh] OR chronic disease[mh] OR clinical trials data monitoring committees[mh] OR cognitive function[tw] OR ec[sh] OR death[mh] OR diffusion of innovation[mh] OR discharge[tw] OR economics, pharmaceutical[mh] OR evidence based practice[mh] OR functional status[tw] OR guideline adherence[mh] OR harm[tw] OR harms[tw] OR health services research[mh] OR health status[mh] OR hospitalization[mh] OR interventions[tw] OR life expectancy[mh] OR longevity[mh] OR models, statistical[mh] OR models, theoretical[mh:noexp] OR morbidity[mh] OR mortality[mh] OR noninferior[tw] OR noninferiority[tw] OR outcome and process assessment[mh] OR outcome[tw] OR outcomes[tw] OR patient compliance[mh] OR postoperative care[mh] OR postoperative complications[mh] OR product surveillance, postmarketing[mh] OR propensity score[tw] OR quality-adjusted life years[mh] OR quality of life[mh] OR recovery of function[mh] OR recurrence[mh] OR relapse[tw] OR remission[tw] OR reoperation[mh] OR risk[tw] OR risk management[mh] OR survival analysis[mh] OR survival rate[mh] OR technology assessment, biomedical[mh] OR trial[ti] OR trials[ti]))) OR clinical effectiveness[tw]) NOT systematic[sb])

### Table A: Defination of timing of RRT

| **Author** | **Early RRT Criteria** | **Late RRT Criteria** |
| --- | --- | --- |
| Bouman 2002 | RRT within 12 h if urine output < 30 ml/h, Cr clearance < 20 ml/min, and mechanical ventilation | Urea > 40 mmol/L or K > 6.5 mmol/L or severe pulmonary edema |
| Durmaz 2003 | Preoperative prophylactic RRT in all patients and postoperative sCr increased >10% | Postoperative sCr increased >50% or urine output |
| Sugahara 2004 | Urine output < 30 ml/h for 3 h or urine output < 750 ml/day | Urine output < 20 ml/h for 2 h or urine output < 500 ml/day |
| Payen 2009 | RRT for 96-h period within 24 h of diagnosis of severe sepsis | Classic indications for RRT |
| Jamale 2013 | Serum urea nitrogen >70 mg/dL and/or creatinine >7 mg/dL | Classic indications for RRT or Uremic nausea and anorexia |
| Combes 2015 | RRT within 24 h of diagnosis of post-cardiac surgery shock | Creatinine >4 mg/dL or preoperative creatinine × 3 or urine output 36 mmol/L or life-threatening hyperkalemia |
| Wald 2015 | sCr increased >200%, urine output < 6 ml/kg within 12 h, or NGAL ≥ 400 ng/ml | K > 6.0 mmol/L or serum bicarbonate < 10 mmol/L or pulmonary edema |
| Gaudry 2016 | RRT within 6 h of diagnosis of KDIGO stage 3 | K > 6.0 mmol/L or PH < 7.15 or pulmonary edema or blood urea nitrogen >112 mg/dL or oliguria >72 h |
| Zarbock 2016 | RRT within 8 h of diagnosis of KDIGO stage 2 | RRT within 12 h of KDIGO stage 3 or no RRT |
| Barbar 2018 | RRT within 12 hours after documentation of failure-stage acute kidney injury | RRT after a delay of 48 hours if renal recovery had not occurred |
| Lumlertgul 2018 | RRT initiation within 6 h after randomization | RRT initiation according to standard indications |

### Table B: Defination of metabolic acidosis and hypotension

| Trials | metabolic acidosis | hypotension |
| --- | --- | --- |
| Durmaz 2003 | Unclear | Not available |
| Jamale 2013 | Unclear | Unclear |
| Combes 2015 | a pH of less than 7.20 | requiring introduction or increase in vasopressors from Day 1 to 30. |
| Wald 2015 | Not available | Unclear |
| Barbar 2018 | a pH of less than 7.15 and a base deficit of more than 5 mmol per liter or a bicarbonate level of 18 mmol or less per liter. | a mean arterial pressure of 55 mm Hg or less and an increase in vasopressor dose or a reintroduction of vasopressors |

### Table C: Sensitivity analyses

| Sensitivity analyses | RR, 95% CI | I^2^ |
| --- | --- | --- |
| Excluding studies published earlier than 2005 | 1.01 [0.91, 1.12] | 0% |
| Excluding studies including fewer than 100 patients | 1.00 [0.90, 1.11] | 0% |
| Excluding studies with non-low risk of bias of each domain |  |  |
| Sequence generation | 1.01 [0.89, 1.15] | 20% |
| Allocation concealment | 1.03 [0.83, 1.28] | 45% |
| Blinding of patients and personnel | Not estimable | Not estimable |
| Blinding of outcome assessors | Not estimable | Not estimable |
| Incomplete outcome data | 0.98 [0.82, 1.16] | 47% |
| Selective reporting | 1.02 [0.92, 1.13] | 0% |
| Other bias | 0.99 [0.83, 1.17] | 48% |
| Excluding one study at a time |  |  |
| Bouman 2002 | 0.99 [0.83, 1.17] | 48% |
| Durmaz 2003 | 1.01 [0.87, 1.17] | 37% |
| Sugahara 2004 | 1.01 [0.90, 1.14] | 13% |
| Payen 2009 | 0.98 [0.82, 1.16] | 47% |
| Jamale 2013 | 0.97 [0.83, 1.14] | 41% |
| Combes 2015 | 0.99 [0.83, 1.18] | 49% |
| Wald 2015 | 0.99 [0.83, 1.19] | 49% |
| Gaudry 2016 | 1.03 [0.88, 1.22] | 40% |
| Zarbock 2016 | 1.00 [0.82, 1.23] | 48% |
| Barbar 2018 | 0.98 [0.81, 1.19] | 48% |
| Lumlertgul 2018 | 0.98 [0.82, 1.19] | 49% |

### Figure A: Risk of bias summary: review authors' judgements about each risk of bias item for each included study.


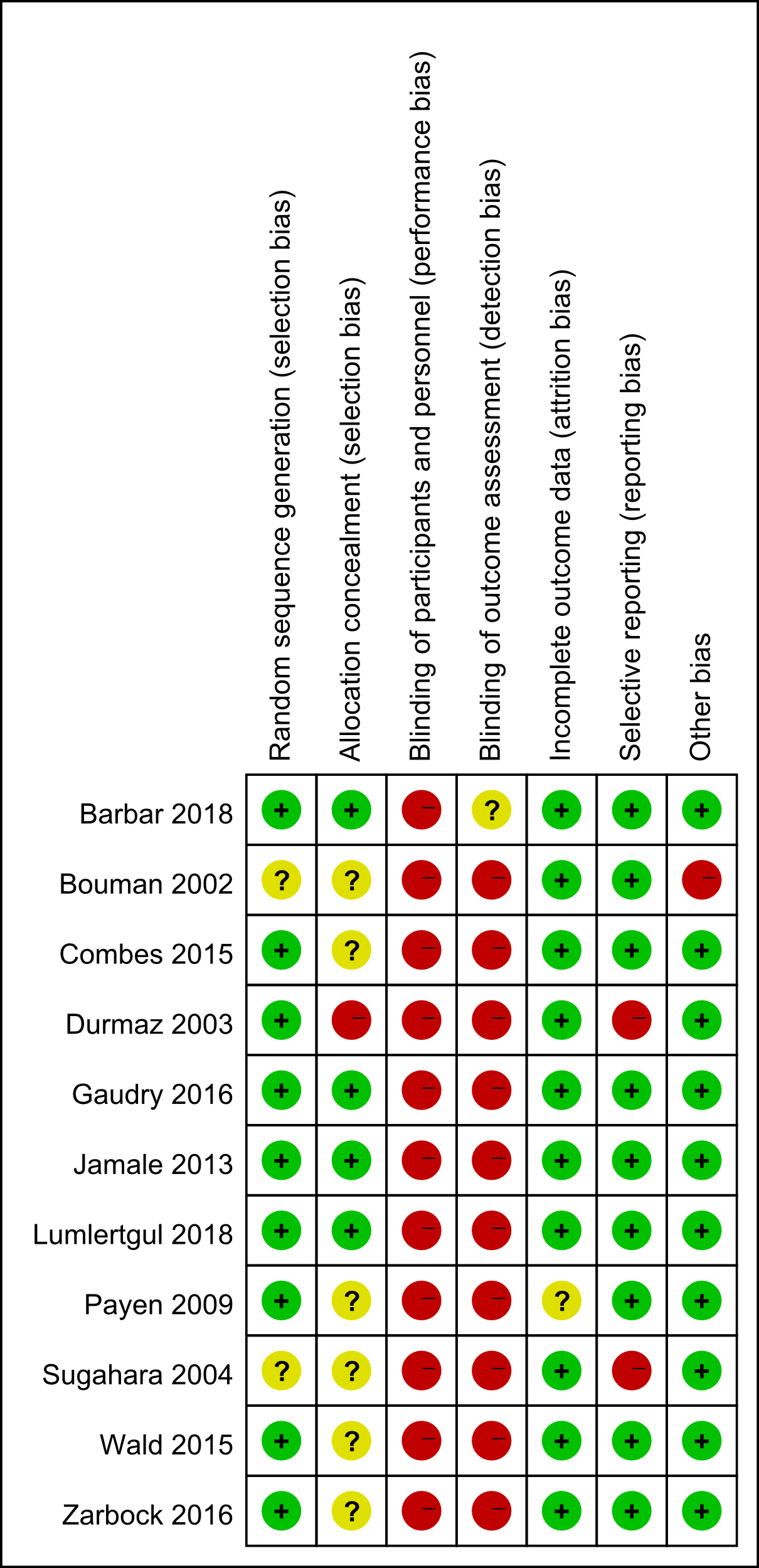


### Figure B: Risk of bias graph: review authors' judgements about each risk of bias item presented as percentages across all included studies


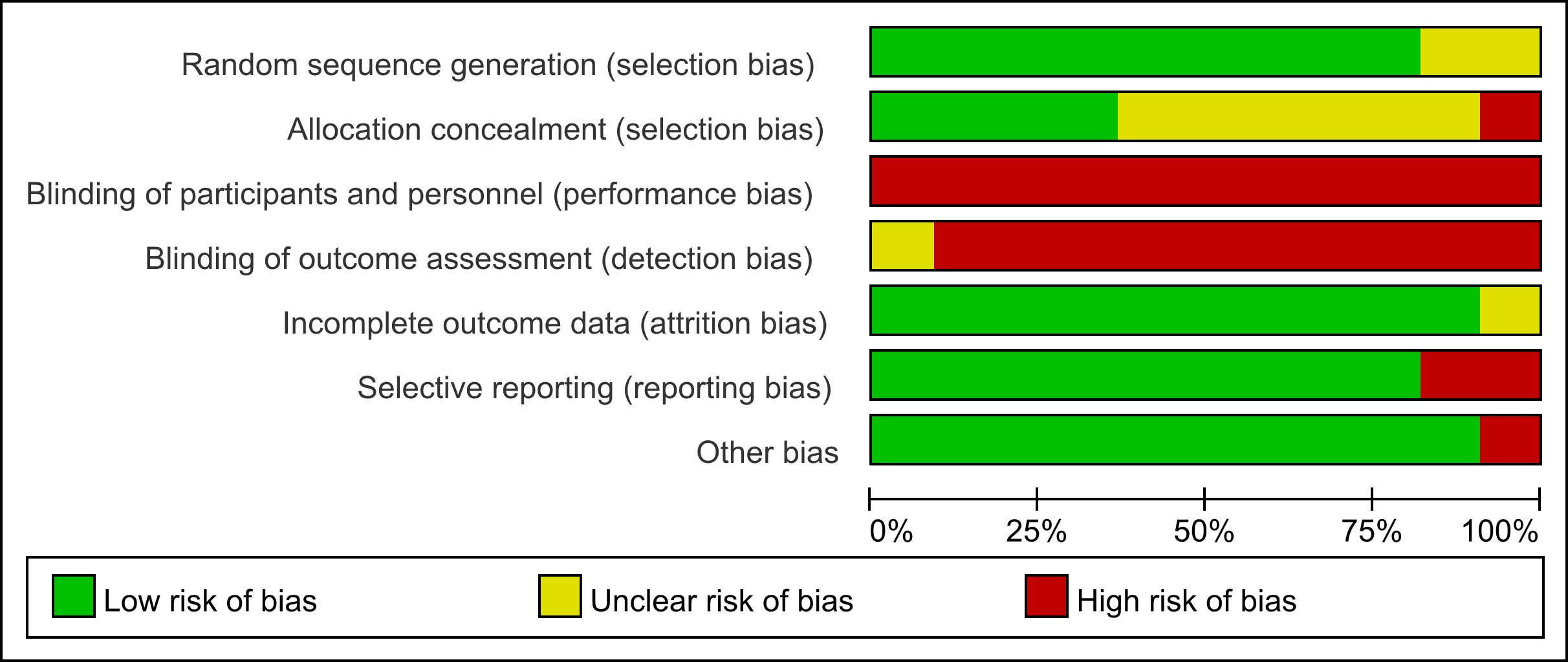


### Figure C: Trial sequential analysis for short-term mortality

###
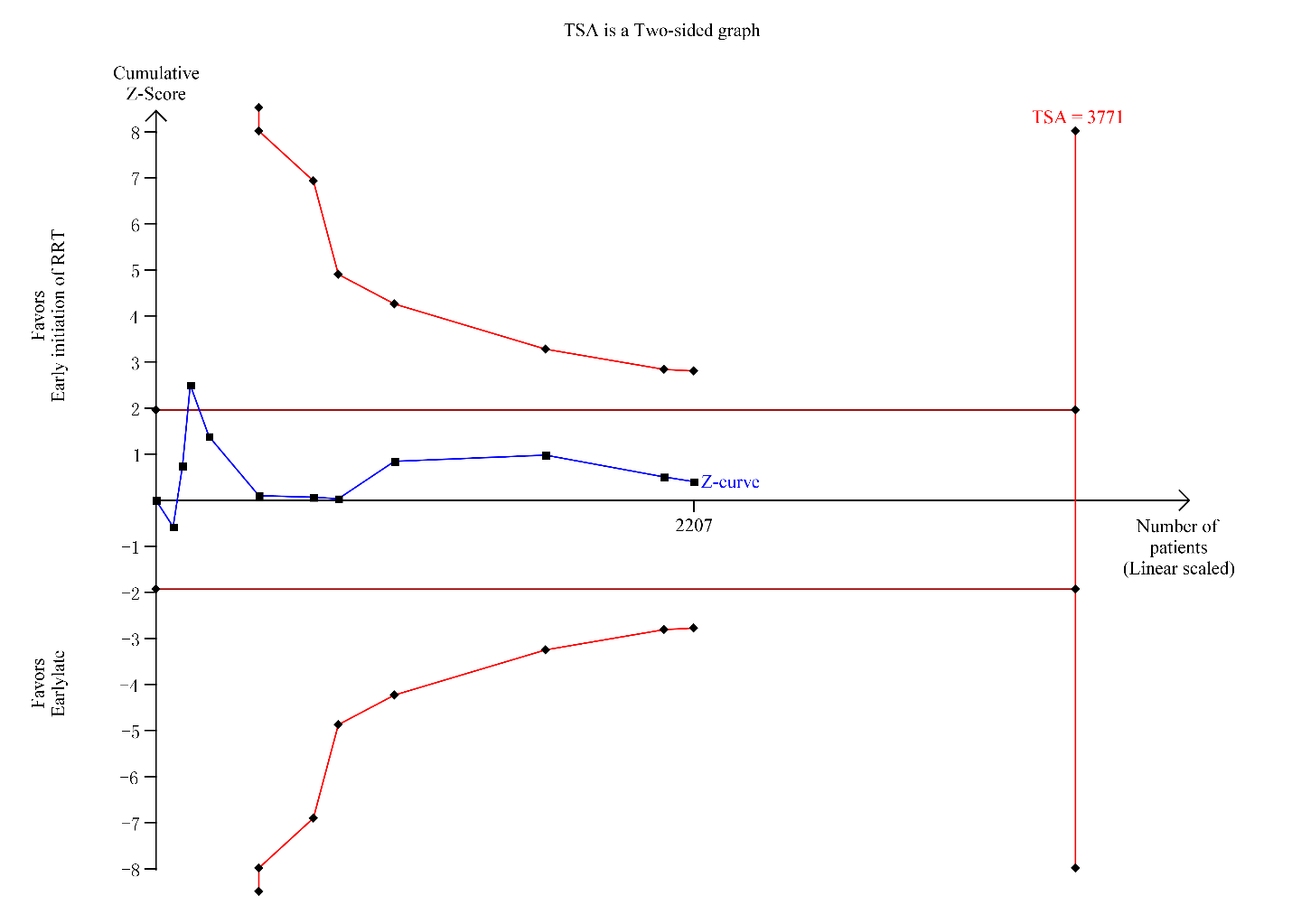


A DIS of 3771 patients was calculated based on an anticipated RRR of 20% (event proportion of 40% in the control arm, α=0.05 (two-sided), β=0.20 (power 80%)). The blue cumulative z-curve was constructed using a random-effects model.

### Figure D: Funnel plot of comparison: early versus late initiation of RRT, outcome: short term mortality.


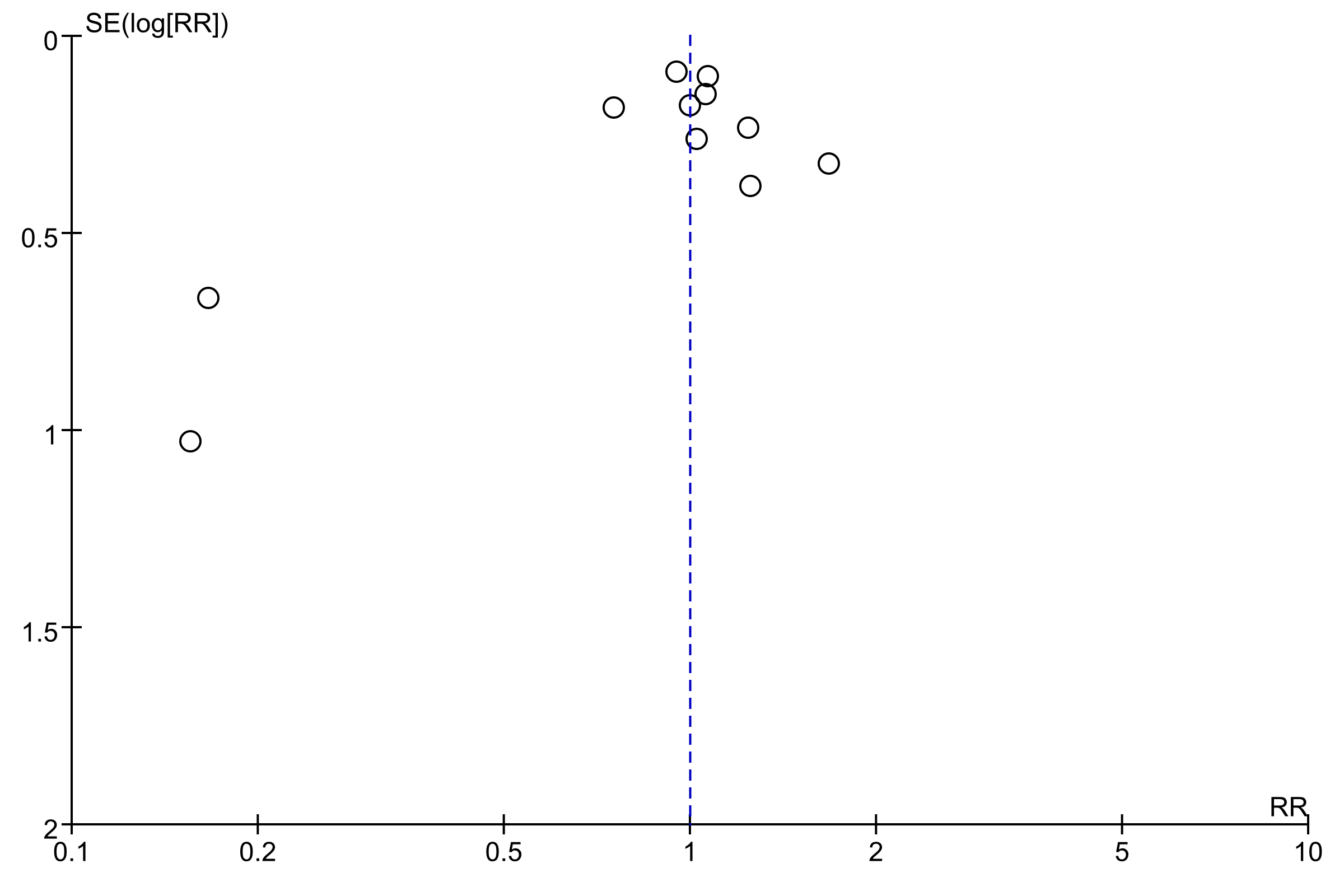


### Figure E: Forest plot for ICU length of stay. df = degrees of freedom, M-H = Mantel-Haenszel.

###
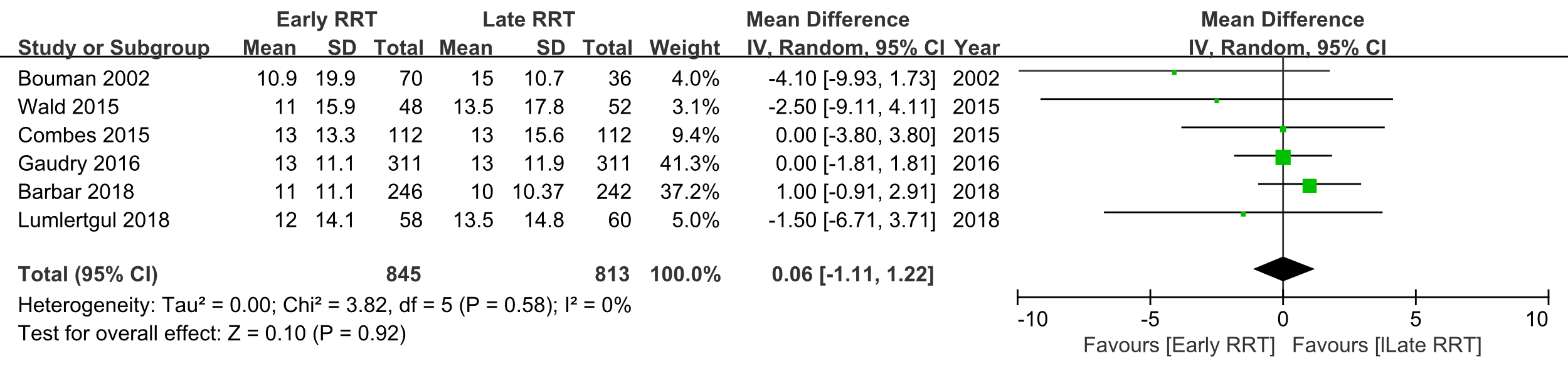


### Figure F: Forest plot for hospital length of stay. df = degrees of freedom, M-H = Mantel-Haenszel.

###
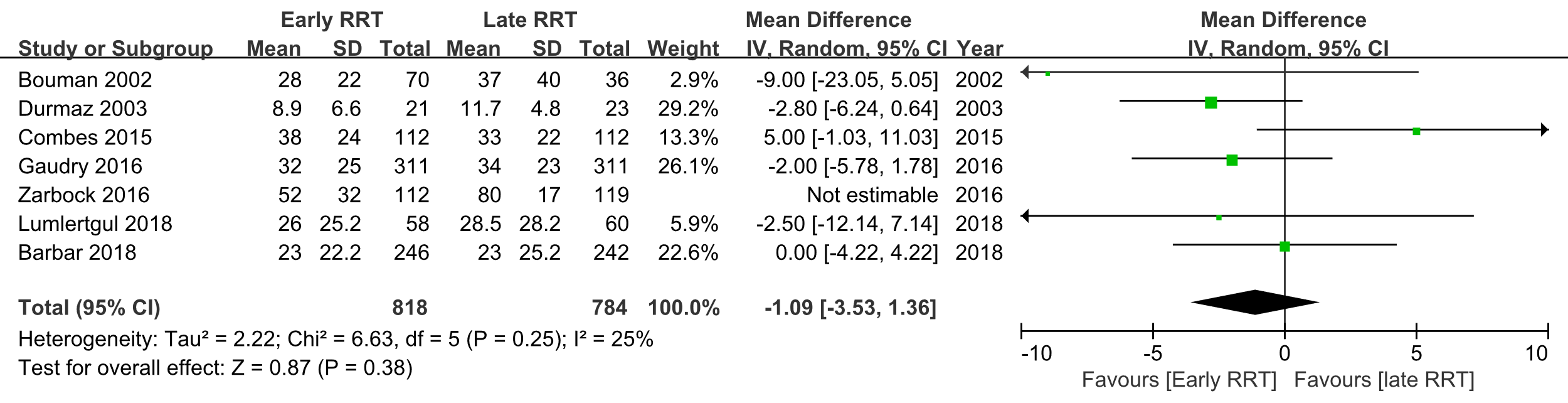


### Figure G: Forest plot for renal function recovery. df = degrees of freedom, M-H = Mantel-Haenszel.


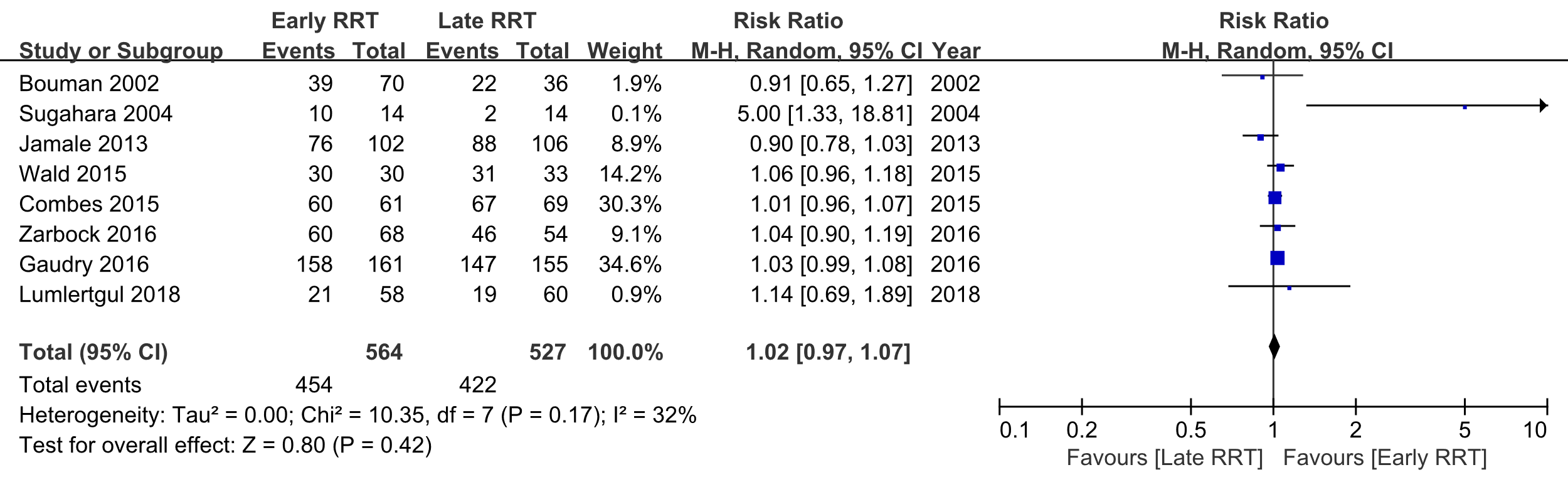


### Figure H: Forest plot for renal replacement therapy dependence. df = degrees of freedom, M-H = Mantel-Haenszel.

###
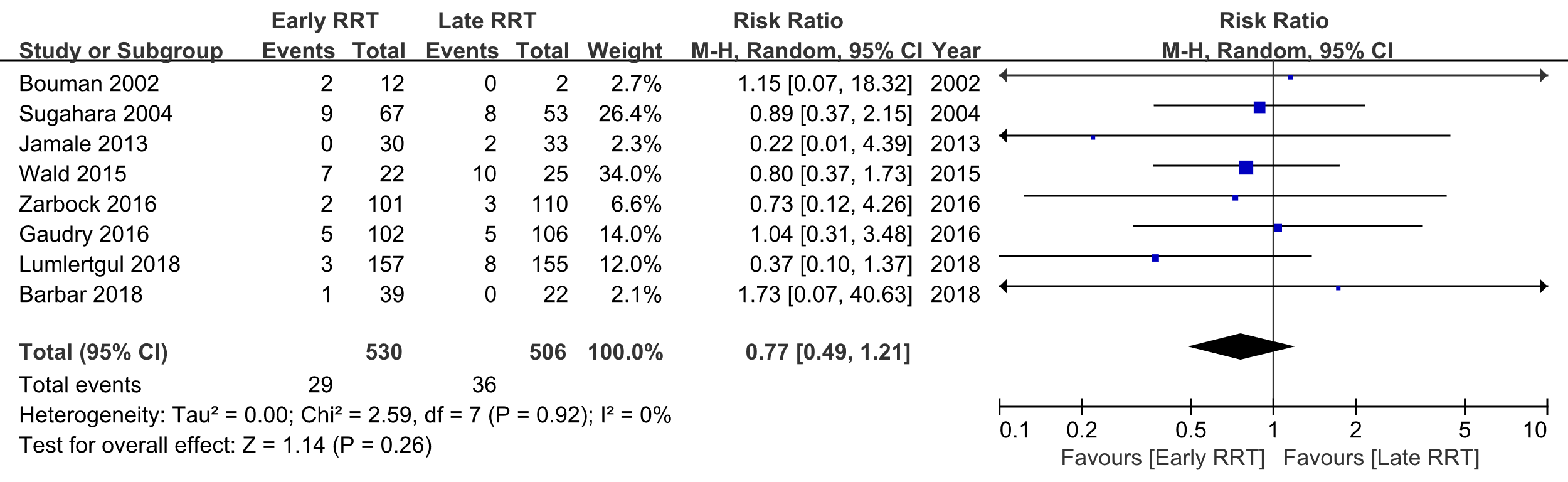


### Figure I: Forest plot for metabolic acidosis. df = degrees of freedom, M-H = Mantel-Haenszel.


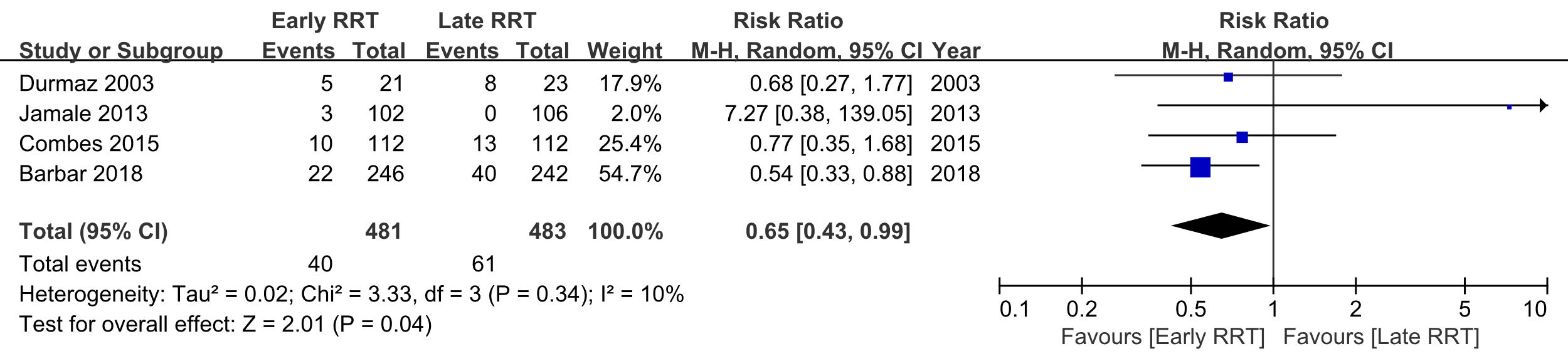


### Figure J: Forest plot for hypotension. df = degrees of freedom, M-H = Mantel-Haenszel.


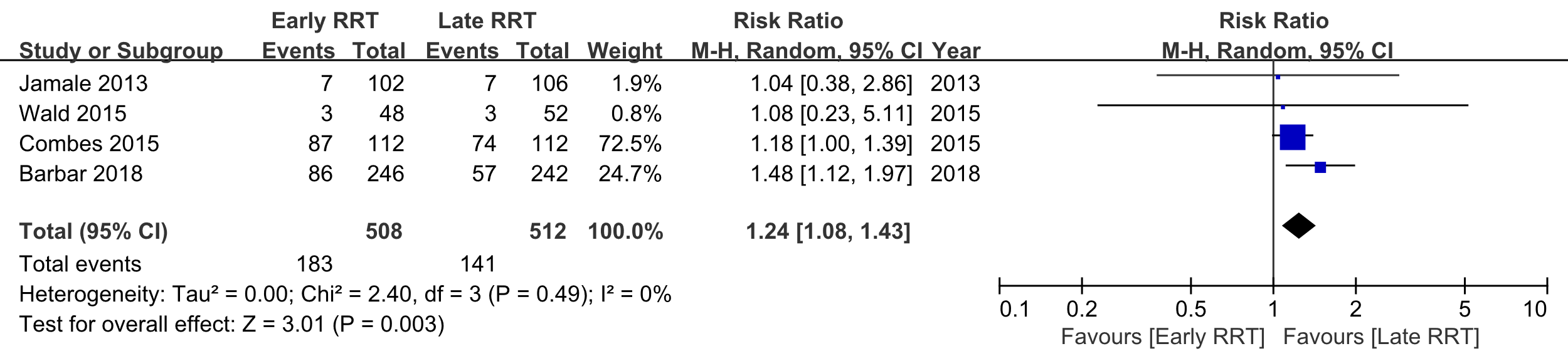

Supplement: S1 File — Material A: Search strategy for Medline; Table A: Definition of timing of RRT; Table B: Sensitivity analyses; Table: Sensitivity analyses; Figure A: Risk of bias summary; Figure B: Risk of bias graph; Figure C: Trial sequential analysis for short-term mortality; Figure D: Funnel plot for short-term mortality; Figure E: Forest plot for length of stay in hospital; Figure F: Forest plot for length of stay in ICU; Figure G: Forest plot for renal function recovery; Figure H: Forest plot for renal replacement therapy dependence; Figure I: Forest plot for metabolic acidosis; Figure J: Forest plot for hypotension. (DOCX) [file pone.0223493.s001.docx]
